# Comparing Medical Term Usage Patterns of Professionals and Search Engine and Community Question Answering Service Users in Japan: Log Analysis

**DOI:** 10.2196/13369

**Published:** 2020-04-13

**Authors:** Kazuya Taira, Taichi Murayama, Sumio Fujita, Mikiko Ito, Kei Kamide, Eiji Aramaki

**Affiliations:** 1 Department of Public Health Nursing Shiga University of Medical Science Otsu, Shiga Japan; 2 Social Computing Laboratory Nara Institute of Science and Technology Ikoma Japan; 3 Yahoo Japan Corporation Tokyo Japan; 4 Division of Health Science Osaka University Osaka Japan

**Keywords:** health knowledge, internet, search engine, community question answering service, information-seeking behavior

## Abstract

**Background:**

Despite increasing opportunities for acquiring health information online, discussion of the specific words used in searches has been limited.

**Objective:**

The aim of this study was to clarify the medical information gap between medical professionals and the general public in Japan through health information–seeking activities on the internet.

**Methods:**

Search and posting data were analyzed from one of the most popular domestic search engines in Japan (Yahoo! JAPAN Search) and the most popular Japanese community question answering service (Yahoo! Chiebukuro). We compared the frequency of 100 clinical words appearing in the clinical case reports of medical professionals (clinical frequency) with their frequency in Yahoo! JAPAN Search (search frequency) logs and questions posted to Yahoo! Chiebukuro (question frequency). The Spearman correlation coefficient was used to quantify association patterns among the three information sources. Additionally, user information (gender and age) in the search frequency associated with each registered user was extracted.

**Results:**

Significant correlations were observed between clinical and search frequencies (*r*=0.29, *P*=.003), clinical and question frequencies (*r*=0.34, *P*=.001), and search and question frequencies (*r*=0.57, *P*<.001). Low-frequency words in clinical frequency (eg, “hypothyroidism,” “ulcerative colitis”) highly ranked in search frequency. Similarly, “pain,” “slight fever,” and “numbness” were highly ranked only in question frequency. The weighted average of ages was 34.5 (SD 2.7) years, and the weighted average of gender (man –1, woman +1) was 0.1 (SD 0.1) in search frequency. Some words were specifically extracted from the search frequency of certain age groups, including “abdominal pain” (10-20 years), “plasma cells” and “inflammatory findings” (20-30 years), “DM” (diabetes mellitus; 30-40 years), “abnormal shadow” and “inflammatory findings” (40-50 years), “hypertension” and “abnormal shadow” (50-60 years), and “lung cancer” and “gastric cancer” (60-70 years).

**Conclusions:**

Search and question frequencies showed similar tendencies, whereas search and clinical frequencies showed discrepancy. Low-clinical frequency words related to diseases such as “hypothyroidism” and “ulcerative colitis” had high search frequencies, whereas those related to symptoms such as “pain,” “slight fever,” and “numbness” had high question frequencies. Moreover, high search frequency words included designated intractable diseases such as “ulcerative colitis,” which has an incidence of less than 0.1% in the Japanese population. Therefore, it is generally worthwhile to pay attention not only to major diseases but also to minor diseases that users frequently seek information on, and more words will need to be analyzed in the future. Some characteristic words for certain age groups were observed (eg, 20-40 years: “cancer”; 40-60 years: diagnoses and diseases identified in health examinations; 60-70 years: diseases with late adulthood onset and “death”). Overall, this analysis demonstrates that medical professionals as information providers should be aware of clinical frequency, and medical information gaps between professionals and the general public should be bridged.

## Introduction

Since the 1990s, the popularization of the internet and personal information devices such as personal computers, smartphones, and tablets has become widespread. Further, opportunities to acquire health information from the web have been increasing. A study examining changes in the basic attributes of information users on the internet in Sweden in 2010 and 2013 showed that the range of users is widening to include younger generations and women [1]. In the United States, 59% of adults were reported to acquire their health information from the internet in 2013 [2]. A survey conducted by the Ministry of Internal Affairs and Communications in Japan in 2015 showed that approximately 80% of people acquired health information via the internet, regardless of gender or age [3]. Moreover, there are many difficulties faced by general users in the process of seeking health information and determining its reliability. In fact, misleading information from the internet could result in serious health hazards [4].

Despite these limitations, health information services have been reported to reduce costs such as medical expenses, to improve production efficiency, and to bring benefits beyond investment [5-7]. The World Health Organization recommended the promotion of such services to member countries from the viewpoint of the quality and safety of medical care, and the possibility of improving access to medical information [8,9]. Taking measures such as the proper management of health information on the web, which is more widely used by the general population, as well as the improvement of services in hospitals and facilities can help promote the management of chronic diseases [10], improve patients’ self-efficacy [11], and support treatment decisions [12]; further, the cost effectiveness for improving the health condition of citizens will be high.

In Japan, eHealth Net developed by the Ministry of Health, Labour and Welfare [13], and the websites of Japanese local governments, national research institutes, and academic societies provide reliable health information. Because professionals create the content of these websites, people believe in the validity and current relevance of the information provided and deem the information to have a reliable level of quality. However, it is difficult to find the necessary information from a wide variety of websites where information is segmented according to expertise. In some countries, the government (or its subsidiary organizations) provides comprehensive services, such as the National Health Service in the United Kingdom and MedlinePlus in the United States; however, there is currently no such health service counterpart in Japan.

Conversely, community question answering (CQA) services can directly answer users’ questions, and websites with user-generated content such as NAVER [14] are very convenient because they can provide information (gathered for easy skimming) on a given topic; however, the trustworthiness of the provided information cannot be guaranteed due to the lack of expertise of the content providers. To address this issue, there is a movement to ensure the reliability of health information provided on such websites created by the private sector through certification systems such as the Health on the Net Foundation Code of Conduct [15].

It is important to evaluate websites that provide medical information, as the general public tends to be confused by technical terms in their search for reliable websites and information with regard to not only understanding available information but also formulating search queries. For example, searching for “my skin itches” may not lead one to the correct website, which might instead be found by searching for “pruritus cutaneous.” It is also necessary for experts who disseminate information to understand these differences when organizing information on websites.

A related study reported that there are not many medical or health-related search queries on the Web, and that the total number of such queries is decreasing compared to the rise in e-commerce search terms [11]. However, another report indicated that health queries account for 4.5% of all searches on two search engines, and that at least 6.75 million health searches are conducted daily [16], indicating that people access a significant amount of health information online.

Further, studies on the quality of health-information websites analyzing search engine rankings and page view statistics demonstrated that English-language Wikipedia is a useful online resource among websites that provide health information [9]. The tools developed to evaluate health information on the internet have also been examined; however, it is still unclear whether or not they are useful because the reliability and validity of many existing tools have not been determined [10].

In addition, studies analyzing people’s search process and search logs have reported that they are unable to find the health or medical information they were seeking and have difficulty in formulating effective queries [17,18]. One study that analyzed logs on the Japanese CQA service Yahoo! Chiebukuro reported that people find it difficult to ask questions, and that they are more likely to be interested in information on various health or disease stages and in the content posted by people with similar experiences [12]. In the search process, symptoms and disease names are searched for alternately, and users tend to experience a sense of anxiety during this process [19-21]. Moreover, adverse health effects may result from self-diagnosis and self-treatment [13,22]; therefore, it is crucial to develop a web environment that does not cause excessive anxiety and health hazards.

Studies comparing search queries between medical professionals and the general public have shown that health care expertise affects users’ query selection and assessment of website quality [23]; furthermore, medical professionals use search engines more often, and spend more time in searching and formulating longer queries [24,25]. Thus, leveraging the search behavior of medical professionals to reformulate queries by general users can help improve their search results [26].

Although the above-mentioned studies attempted to improve users’ search results to help the general public access more relevant medical information [23,26,27], it is also important for medical professionals to understand users’ medical information needs in order for them to provide not only reliable but also accessible medical information on the internet.

Therefore, the aim of the current study was to clarify the difference in the frequently used words by medical professionals and by general internet users; for this purpose, we used data from one of the largest Japanese search engines and a CQA service primarily involving Japanese-speaking users. The results of this study will be useful in identifying the unmet needs of general internet users and in helping health professionals provide medical information tailored to general users.

## Methods

We analyzed the query/question logs of the search engine “Yahoo! JAPAN Search” and the CQA service “Yahoo! Chiebukuro” (the Japanese counterpart of Yahoo! Answers), which were provided by the Yahoo Japan Corporation. Data were acquired from the earliest available date for each service up to August 2018: September 2013 to August 2018 for “Yahoo! JAPAN Search” and November 2005 to August 2018 for “Yahoo! Chiebukuro.”

We compared the frequency of 100 clinical words appearing in a clinical case search log by medical professionals (clinical frequency) with their frequency in the search logs of Yahoo! JAPAN Search (search frequency) and in the posted questions of Yahoo! Chiebukuro (question frequency). The top 100 words were used for each word frequency category, as this is frequently used by medical staff [28]. The frequency of words in the ~45,000 case reports of the Japan Science Foundation was used for calculating the word use frequency of medical professionals. We analyzed electronic medical records and discharge summaries written by medical professionals from cooperating hospitals, and extracted 100 frequently used words from the MANBYO Dictionary [29], containing not only disease names specified by international standards such as the ICD 10 Standard Disease Name Master (V 4.04 Revised April 1, 2018) but also all symptoms, including abbreviations and the English names of diseases. Approximately 1.6 million words related to symptoms and diseases were extracted from about 290,000 documents, including approximately 363,000 frequently occurring words related to symptoms and diseases merged with words in the ICD 10. These words are hereafter referred to as “clinical frequency 100 words” [28].

To assess the validity of limiting the study to 100 clinical words, we employed three medical and nursing professionals to assist in determining the 100 clinical words. The 100 most frequent clinical words included both generally common words such as “fever,” “diabetes,” and “hypertension,” and medical specialty words such as “multiple myeloma” and “dermatomyositis.” Therefore, in clarifying the medical information gap between medical professionals and the general public, we concluded that the use of 100 words was appropriate from the viewpoint of visibility.

We counted the frequency of the clinical frequency 100 words in the query logs of “Yahoo! JAPAN Search” (hereafter referred to as “search frequency 100 words”) and about 16 million questions posted in the category of “Health, Beauty, and Fashion” of “Yahoo! Chiebukuro” (hereafter referred to as “question frequency 100 words”). In calculating the question frequency 100 words, a morphological analysis was performed on the character information data with MeCab using the Mecab-ipadic-Neologd dictionary [30], and the occurrence frequency was counted. Thereafter, the Spearman correlation coefficient was used to analyze the association patterns among the three frequencies.

We also performed a qualitative analysis of words with a low ranking in the clinical frequency 100 words (particularly the top 10 words) but a high ranking in the search and question frequency categories. That is, words that were not frequently used by medical staff but are often searched on the web.

In addition, for the search frequency 100 words, the retrieval history associated with registered user information was extracted, and descriptive statistics by gender and age were confirmed. For aggregation by gender and age, the weighted average was calculated, and words that were out of the range of an average value with 2 SD were extracted as unique words for each age and gender:

Gender: Σ(*_k_*_=1_^100^ [–1 × Man_N_*_k_* + 1 × Woman_N_*_k_*]/Man_N_*_k_* + Woman_N_*_k_*)/100, where N is the number of searches.

Age: Σ*_k_*_=1_^100^ ([10s_N_*_k_* × 10 + 20s_N_*_k_* × 20 + 30s_N_*_k_* × 30 + 40s_N_*_k_* × 40 + 50s_N_*_k_* × 50 + 60s_N_*_k_* × 60]/[10s_N_*_k_* + 30s_N_*_k_* + 40s_N_*_k_* + 50s_N_*_k_* + 60s_N_*_k_*])/100, where Xs_N_ is the number of searches according to X decade of age.

The data analyzed in this study were based on the sum of search results for each of the following devices: personal computer, tablet, and smartphone. The analysis was performed using Python ver. 3.6.5 (Python Software Foundation [31]).

## Results

According to StatCounter Global Stats [32], “Yahoo! JAPAN Search” accounted for 28.06% (range 15.82%-40.49%) of the Japanese search engine share in all devices (personal computer, tablet, and smartphone) from 2009 to 2018. Globally, this share is 3.28% (range 1.61%-6.01%) on average. Therefore, most users of this search engine are Japanese, and it was thus considered to be a useful source for understanding the general search situation in Japan.

**Figure 1:**
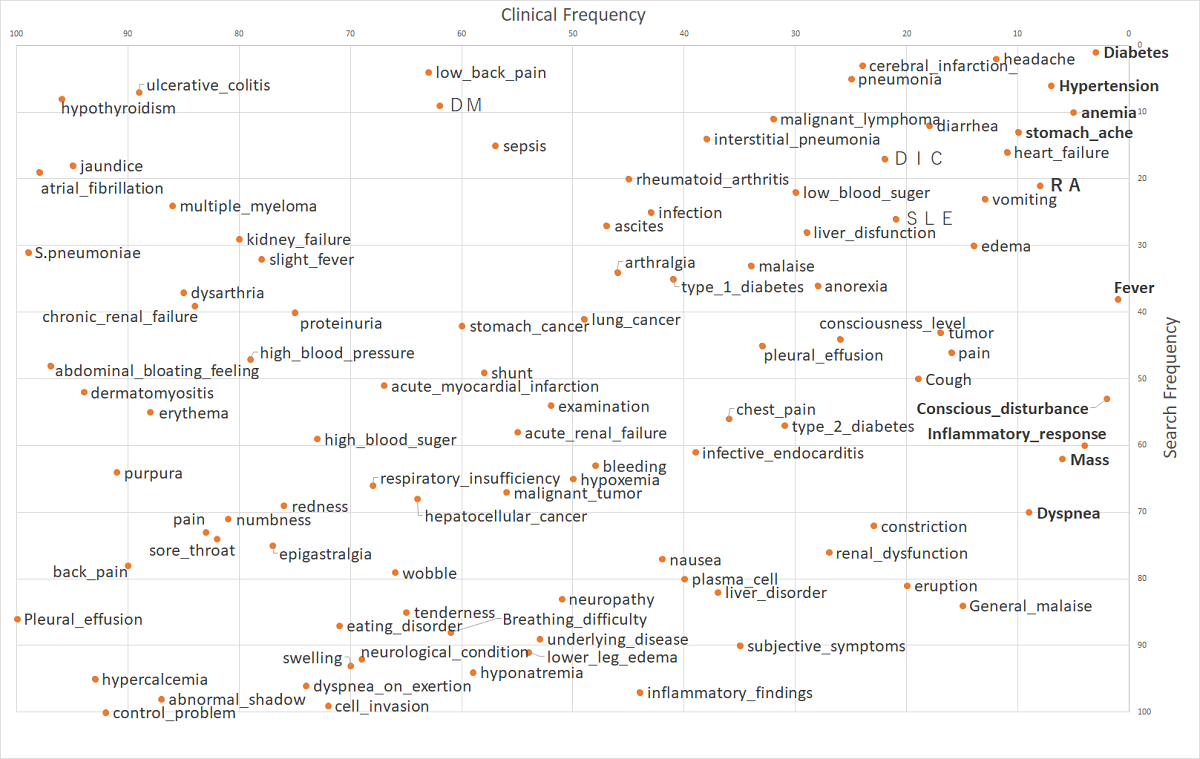
Rank plot of clinical and search frequencies in the search engine.

**Figure 2:**
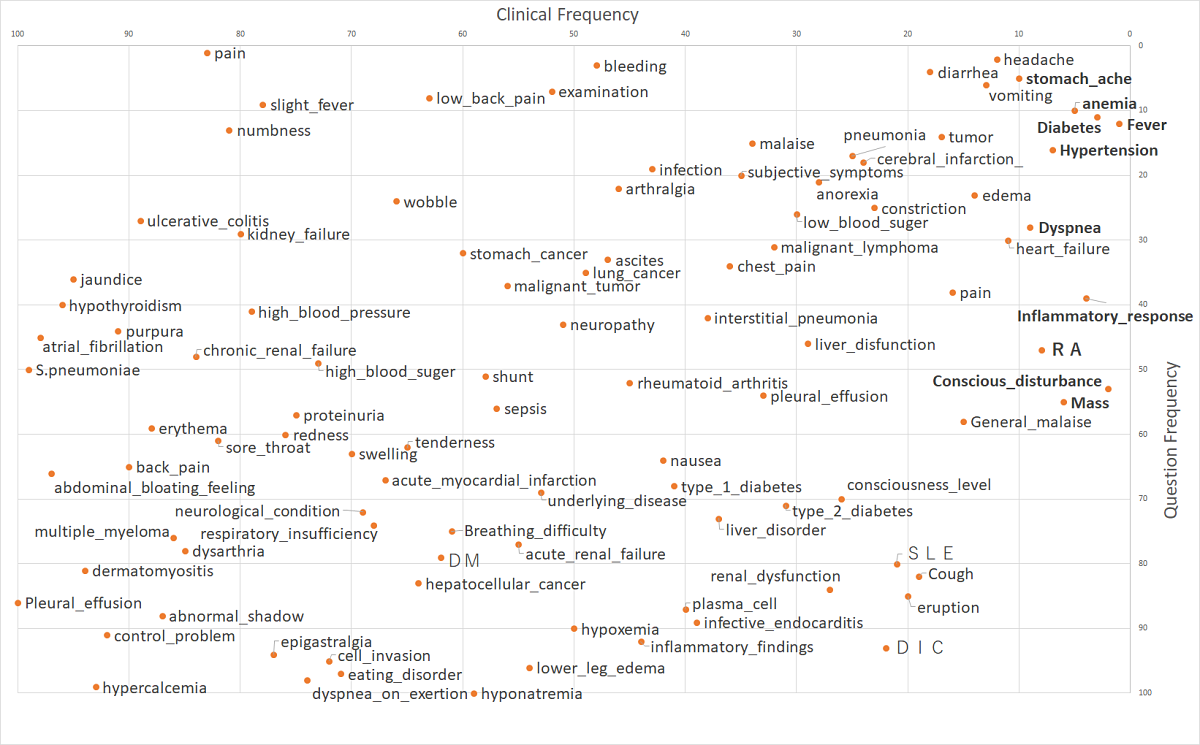
Rank plot of clinical and question frequencies in the community question answering service.

**Figure 3:**
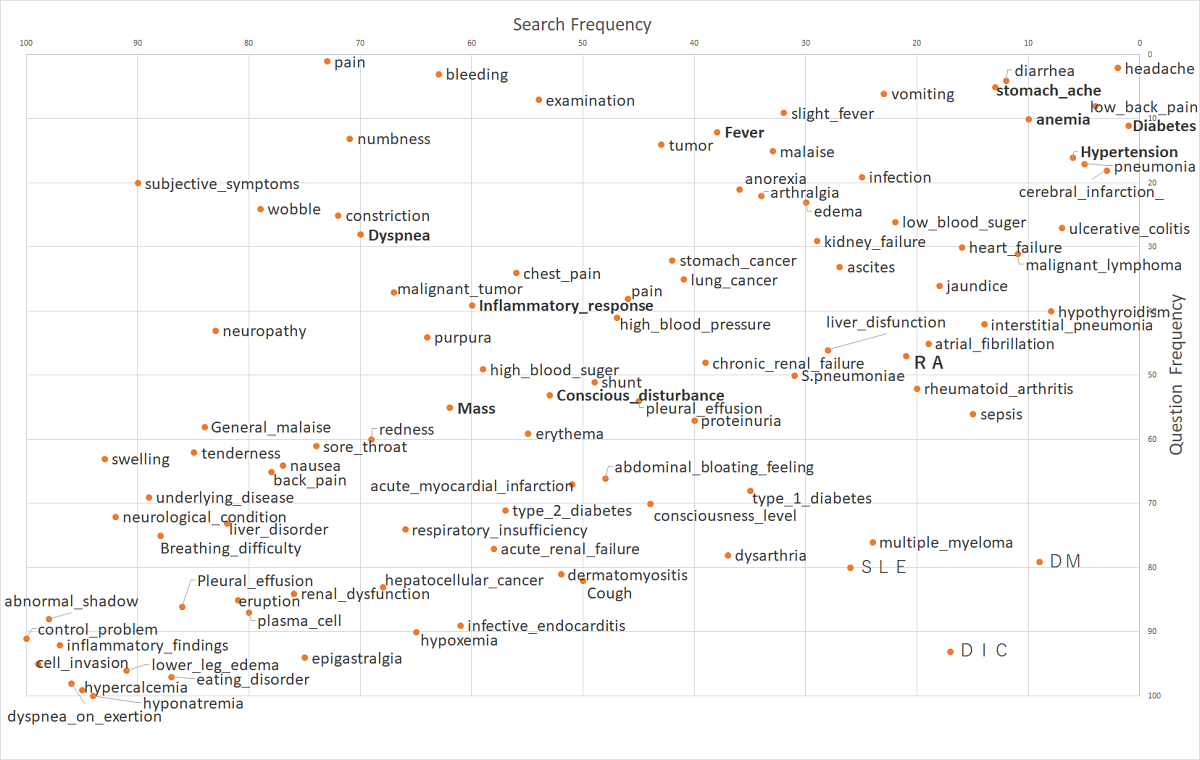
Rank plot of question frequency in the community question answering service and search frequency in the search engine.

The results of the correlation analysis of the clinical frequency 100 words, search frequency 100 words, and question frequency 100 words are shown in Table 1, and the respective plot diagrams are shown in Figures 1-3 (also see Multimedia Appendix 1). Note that in Figures 1-3, only words in all uppercase letters were searched for using the Latin alphabet, not in Japanese, and boldface words indicate the top 10 words with regard to clinical frequency.

**Table 1:** Correlation coefficients of the search rank of each platform.

Frequency type	Clinical Frequency	Search Frequency	Question Frequency
Clinical Frequency	1.000	0.290, *P=*.003	0.337, *P*=.001
Search Frequency (Search Engine)	—^a^	1.000	0.569, *P*<.001
Question Frequency (CQA^b^ service)	—	—	1.000

^a^Not applicable.

^b^CQA: community question answering.

There was a weak correlation between clinical frequency 100 words and search frequency 100 words (Table 1). Qualitative analysis showed that diseases that are the main cause of death in Japan and the lifestyle diseases that cause them such as diabetes, hypertension, headache, anemia, abdominal pain, heart failure, and cerebral infarction ranked highly for both frequencies. By contrast, words for the evaluation of a patient’s condition that are often used by medical professionals, such as ascites retention, hypercalcemia, poor control, and back pain, had a low ranking in both clinical and search frequency 100 words. Words with a low ranking in clinical frequency 100 words and high ranking in search frequency 100 words—that is, the frequency of use by medical staff is relatively low but that of the general public in searches is high—included thyroid function degeneration, ulcerative colitis, jaundice, atrial fibrillation, multiple myeloma, and renal failure.

A weak correlation was also found between clinical frequency 100 words and question frequency 100 words (Table 1). Qualitative analysis showed that words related to symptoms such as headache, abdominal pain, diarrhea, vomiting, anemia, diabetes, and fever were among the top-ranked words for both categories, whereas words such as ascites retention, hypercalcemia, poor control, abnormal shadow, and dermatomyositis ranked lower. Words that were ranked lower in clinical frequency 100 words and higher in question frequency 100 words—that is, the frequency of use by medical staff is relatively low but the question frequency in “Yahoo! Chiebukuro” is high—included pain, slight fever, numbness, ulcerative colitis, and renal failure.

Finally, a moderate correlation was found between search frequency 100 words and question frequency 100 words (Table 1). The top words in both frequencies were headaches, lower back pain, diarrhea, abdominal pain, anemia, and diabetes, whereas the words that ranked lower in both frequencies were hyponatremia, cell invasion, dyspnea on exertion, high anemia, lower edema, inflammatory findings, and poor control. Furthermore, among the words with a large divergence between search and question frequency 100 words, those with a high ranking in question frequency 100 words were subjective symptoms, pain, numbness, and wandering, while those with a high ranking in search frequency 100 words were DM (diabetes mellitus), DIC (disseminated intravascular coagulation), and SLE (systemic lupus erythematosus).

Considering the top 10 words in bold type in Figures 1-3, the same trend was observed as found for the top 100 words. The words used by medical professionals when managing patients’ medical conditions, such as conscious disturbance, inflammatory response, and dyspnea, were found less frequently in both search and question frequencies.

The search ratio by gender for search frequency 100 words was 44.4% (SD 8.7%) for men and 54.6% (SD 9.2%) for women; thus, the search rate was higher for women. Likewise, the weighted average according to gender (men coded as –1 and women coded as +1) was 0.1 (SD 0.1), showing a tendency toward greater searching by women (Table 2). Moreover, the search ratios by age were 2.9% (SD 1.5%) in the 10-20 years age group, 24.3% (SD 8.6%) in the 20-30 age group, 26.0% (SD 4.9%) in the 30-40 age group, 24.6% (SD 5.2%) in the 40-50 age group, 13.9% (SD 4.1%) in the 50-60 age group, and 7.3% (SD 3.4%) in the 60-70 age group; thus, those in the 20-50 age group had the highest search rates. The weighted average was 34.5 (SD 2.7) years (Table 2).

To extract words specific to each gender or age, we searched for words whose weighted average value was larger or smaller than the mean (2 SD), which are summarized in Table 3 and Table 4, respectively.

**Table 2:** The number of search words by weighted average (WA) by gender and age.

WA		Number of search words
**WA by gender^a^**		
	WA<–0.3	2
	–0.3≤WA<–0.2	2
	–0.2≤WA<–0.1	5
	–0.1≤WA<0	14
	0≤WA<0.1	22
	0.1≤WA<0.2	28
	0.2≤WA<0.3	18
	0.3≤WA<0.4	7
	WA≥0.4	1
**WA by age (years)**		
	WA<28	1
	28≤WA<30	2
	30≤WA<32	19
	32≤WA<34	23
	34≤WA<36	26
	36≤WA<38	14
	38≤WA<40	13
	WA≥40	1

^a^Man, –1; Woman, +1.

**Table 3:** Search rate by gender and gender-specific queries.


Gender	Mean (SD)	High Frequency	Low Frequency
Men	44.4 (8.7)	RA^a^, DM^b^	None
Women	54.6 (9.2)	None	RA, DM

^a^RA: rheumatoid arthritis.

^b^DM: diabetes mellitus.

**Table 4:** Search rate by age and age-specific queries.

Age (years)	Mean (SD)	High Frequency	Low Frequency
10-20	2.9 (1.5)	dyspnea, stomachache, subjective symptoms, plasma cell	None
20-30	24.3 (8.6)	plasma cell, inflammatory findings, hypoxemia, cell invasion, dyspnea on exertion	None
30-40	26.0 (4.9)	diabetes mellitus, jaundice	high blood pressure
40-50	24.6 (5.2)	abnormal shadow	plasma cell, inflammatory findings, hyponatremia, cell invasion, hypercalcemia
50-60	13.9 (4.1)	rheumatoid arthritis, high blood pressure, abnormal shadow	inflammatory findings
60-70	7.3 (3.4)	interstitial pneumonia, lung cancer, stomach cancer, high blood pressure, atrial fibrillation, *Streptococcus pneumoniae* infection	None

## Discussion

### Principal Findings

We found a moderate correlation between search and question frequency 100 words, whereas clinical frequency 100 words was only weakly correlated with the other two frequencies. Therefore, the words that are frequently used by medical professionals may differ from words used by general users in search engines or when consulting CQA services. In addition, although the content searched in the search engine and in the CQA service was similar, they also showed unique characteristics.

In qualitative analysis, diabetes and hypertension were recognized as words with a high ranking in all frequency categories. Regarding differences, search frequency 100 words showed a slightly higher frequency for disease names such as heart failure and cerebral infarction, and question frequency 100 words showed a slightly higher frequency for more symptomatic names such as headache, diarrhea, and vomiting.

The characteristics of words that are less frequently used by medical professionals but are frequently searched on the internet by general users differed in both search frequency 100 words and question frequency 100 words. For example, in search frequency 100 words, diseases such as hypothyroidism (rank 8) and ulcerative colitis (rank 7) were common, whereas in question frequency 100 words, symptoms such as pain (rank 1), slight fever (rank 9), and numbness (rank 13) were more typical. Related to this finding, Zhang [12] claimed that questions in the CQA service correspond to the disease stage of the user. The results of the present study suggest that, based on the characteristics of the CQA service, people with specific concrete worries and consultation needs more frequently use the CQA service than searching by queries. Therefore, we consider that the top words in search frequency 100 words are conceptual, whereas the top words used in question frequency 100 words are related to more specific symptoms. Studies on the query logs of both experts and the general public have reported conflicting results, with some indicating that the main focus of searches is on symptoms rather than diseases [20], while others suggesting that the main focus is on diseases rather than symptoms [25]; however, differences may arise depending on whether the search is done on a search engine or through a question on a CQA service.

In addition, words with high search frequencies and question frequencies included designated intractable diseases such as “ulcerative colitis”, with an incidence of less than 0.1% in Japan. Thus, information on diseases that affect a large population of patients is not necessarily high, and factors such as the age at which the disease is likely to develop, severity of the symptoms, prognosis in terms of survival, and presence or absence of a treatment method may also be relevant. Despite the fact that ulcerative colitis is designated as an intractable disease, it does not directly influence survival, and even if remission is achieved, a complete cure is not possible. In fact, the specific questions posted in Japanese on Yahoo! Chiebukuro included the following: “Can you play soccer with ulcerative colitis?,” “Can I become a firefighter or a police officer even if I have ulcerative colitis?,” “Can’t ulcerative colitis be cured completely? It is not a remission but a complete cure,” “Ulcerative colitis doesn’t cause death, does it?? My parent has the disease. I’m very worried. Someone please answer m (* _ _) m! [conventional notation for a bowing gesture].”

General users seek to obtain health information from the internet for various purposes, such as interacting with people who have the same experience [33], looking for advice [34], and understanding the diagnosis [20]. As represented by the words “ulcerative colitis” and “hypothyroidism,” people who are not completely cured but are in remission may be more likely to have such purposes, but because of the low risk to life, such diseases and purposes may be given a lower priority by medical professionals as medical information providers. Since there is generally less information available for minor diseases, it is important to value user-oriented information needs and not only information based on major diseases or professional judgment.

Regarding gender in search frequency 100 words, abbreviations used by medical professionals such as “RA” and “DM” ranked at the top of gender-specific words. However, when the details of the search related to these words and the words that were searched together were examined, other words with similar spelling such as “JRA” (Japan Racing Associations), “ZARA” (a fashion brand name), and “DMM” (a company name) were also found. Therefore, careful interpretation of alphabetical abbreviations is necessary. Nevertheless, there were no specific queries according to gender, but the overall proportion of females searching was high. These results are consistent with previous studies [35]. In Japan, the proportion of men participating in child rearing and nursing care has also increased, but as women still carry out many of these traditional roles, it was predicted that women are not only searching for themselves but also for their family’s health-related problems.

Regarding age in search frequency 100 words, for people in their 40s and 50s who are expected to have an increasing number of diagnoses of diseases by medical examination, words such as abnormal shadow and hypertension were highly ranked, whereas words such as stomach and lung cancer, atrial fibrillation, interstitial pneumonia, and pneumococcus ranked higher for those in their 60s. Therefore, there may be a connection between diseases whose prevalence increases with age and health information needs. However, it was somewhat difficult to interpret words such as plasma cells, cell wetness, hypoxemia, and exercise dyspnea, which were extracted as specific words by users in their 20s and 30s. More detailed examination of the searches related to these words and the words that were searched together showed that “nursing” was searched together with these words or long sentences, such as case examples used as tasks for nursing students, which suggested that many searches by nursing students were likely included in this category. However, these words are all related to “cancer” as well as cancer treatment of the adolescent and young adult generation in Japan in recent years. Therefore, we believe that further analysis is necessary to determine the detailed trends of the search tendency of each age group.

### Strengths and Limitations

This study has several limitations. First, since the analysis was limited to the top 100 clinical frequency words, infrequent words used in search engines and CQA services were also included, which may have confounded the correlation analysis between the three word frequencies. Clinical frequency 100 words are centered on general medical terms and do not include words used by medical personnel in psychiatry, dermatology, obstetrics, and gynecology. Second, the log data analyzed in this study are biased toward Japanese users and cannot be generalized globally, and the relationship between the incidence of disease and the information needs of the general population has not been fully explained; thus, further research is needed.

Nevertheless, this study is significant in that the findings reveal that the frequency of some words differed between clinical and search/question frequencies. Further, compared to search engines, CQA data contained more words about symptoms than diseases. Although no clear causal relationship can be established, the number of diseases may be high, at least depending on the nature of the disease. In addition, more women were found to search for medical information on the internet. Lastly, our analysis highlights that the medical information needs differ according to age group.

### Conclusion

In conclusion, when providing medical information on the internet in Japan, medical professionals as information providers should be aware of clinical frequency, and medical information gaps between professionals and the general public should be bridged. Moreover, such information should take into account users’ age and gender, as well as the delivery format of the website and CQA service.

## Appendix

Multimedia Appendix 1 Number of searches and rank of Top 100 words with three types of frequency.

## Abbreviations

CQA: community question answering
DM: diabetes mellitus
DIC: disseminated intravascular coagulation
RA: rheumatoid arthritis
SLE: systemic lupus erythematosus
