# Supplementary figures and images for: Comparing Medical Term Usage Patterns of Professionals and Search Engine and Community Question Answering Service Users in Japan: Log Analysis
Source: J Med Internet Res. 2020 Apr 13;22(4):e13369. doi: 10.2196/13369 (PMC7186863; doi:10.2196/13369)

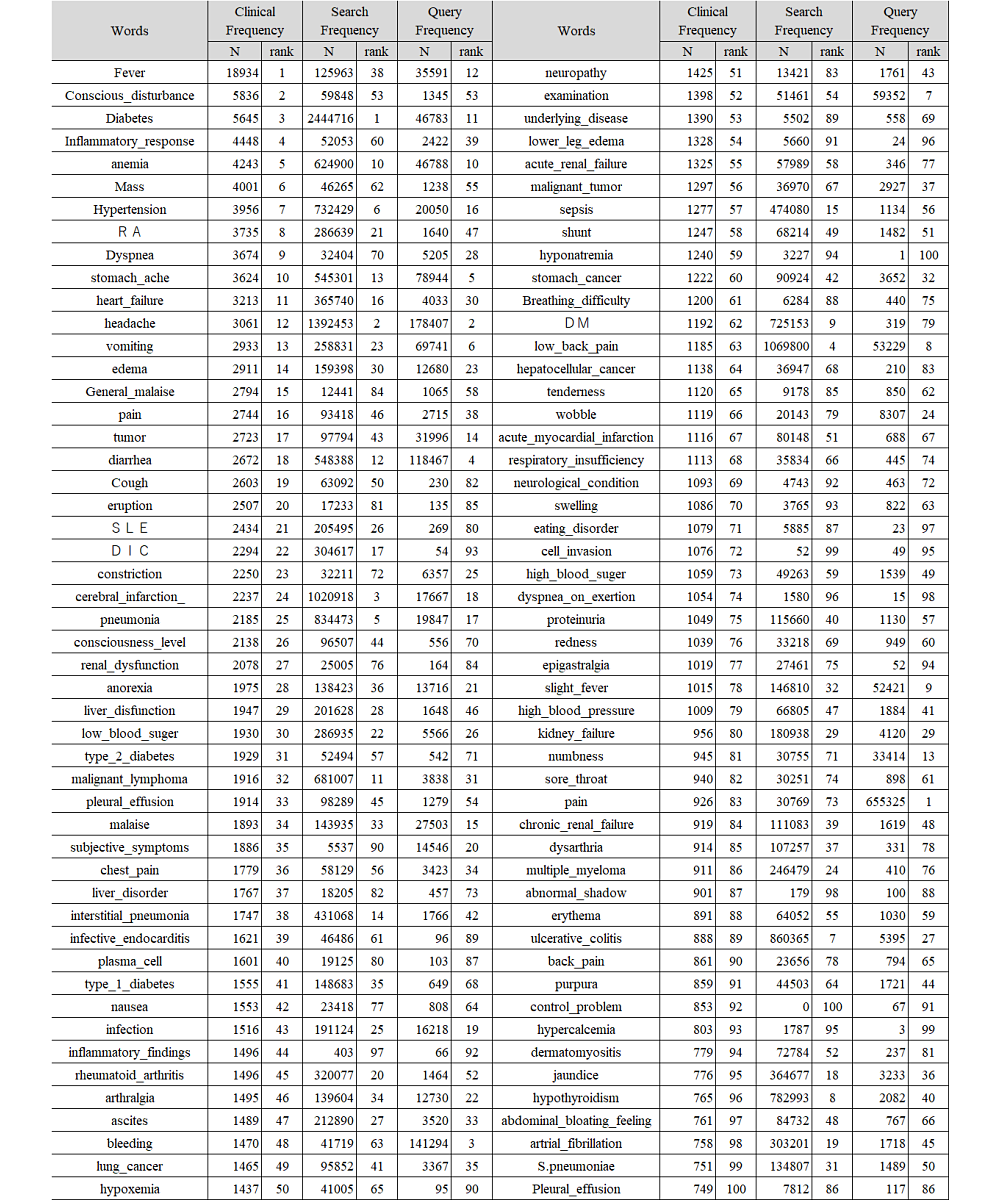

Supplement: Multimedia Appendix 1 [file jmir_v22i4e13369_app1.png]
